# Putative Nickel-Dependent Anaerobic Carbon Monoxide Uptake Occurs Commonly in Soils and Sediments at Ambient Temperature and Might Contribute to Atmospheric and Sub-Atmospheric Carbon Monoxide Uptake During Anoxic Conditions

**DOI:** 10.3389/fmicb.2022.736189

**Published:** 2022-03-24

**Authors:** Amber N. DePoy, Gary M. King

**Affiliations:** Department of Biological Sciences, Louisiana State University, Baton Rouge, LA, United States

**Keywords:** carbon monoxide, anaerobic, soil, sediment, microbial community, diversity, thermophilic

## Abstract

Carbon monoxide (CO) occurs naturally in the atmosphere where it plays a critical role in tropospheric chemistry. Atmospheric CO uptake by soils has been well documented as an important CO sink and has been attributed to a group of aerobic bacteria that possess a molybdenum-dependent CO dehydrogenase (Mo-CODH). CO can also be oxidized by obligate Ni-dependent anaerobes (Ni-COX) that possess nickel-dependent CODHs (Ni-CODH) but relatively little is known about their ecology or their potential to contribute to CO dynamics within soils and sediments or to soil-atmosphere CO exchanges. Results from a series of assays undertaken with diverse soils and sediments and CO concentrations of 10 ppm and 25% with incubation temperatures of 10, 25, and 60°C revealed anaerobic uptake rates with 10 ppm CO that were comparable to those measured under oxic conditions; further, anaerobic CO uptake occurred without a lag and at atmospheric and sub-atmospheric CO concentrations. Assays with 25% CO revealed previously undocumented activity at 10°C and showed extensive activity at 25°C. Results from prior studies with isolates and soils suggest that anaerobic uptake at both 10 ppm and 25% CO concentrations might be attributed to Ni-COX. Collectively the results considerably expand the ecological range for Ni-COX and indicate that they could play previously unsuspected roles in soil CO dynamics.

## Introduction

Aerobic CO-oxidizing bacteria (Mo-COX) occur ubiquitously in soils where they play an important role in the atmospheric CO budget (King, 1999). They possess a form I molybdenum-dependent CO dehydrogenase (Mo-CODH; King and Weber, 2007) and are comprised of multiple phyla, most prominently Actinobacteria and Proteobacteria but also Acidobacteria, Bacteroidetes, Chloroflexi, Crenarchaeota, Euryarchaeota, and Firmicutes (King and Weber, 2007; Cordero et al., 2019). Molecular oxygen is their preferred oxidant but some Mo-COX can oxidize CO anaerobically using nitrate as an electron acceptor (Frunzke and Meyer, 1990; King, 2003b,^2006^). Nonetheless, nitrate-coupled anaerobic CO oxidation by Mo-COX has not be demonstrated for ambient CO concentrations (King, 2003b,^2006^), which calls into question their activity under anaerobic conditions *in situ*.

In contrast, obligately anaerobic CO oxidizers use a well-characterized nickel-dependent CODH [Ni-CODH; (Ragsdale, 2004; Techtmann et al., 2011)]. Ni-dependent CO-oxidizing bacteria (Ni-COX) have been isolated primarily as thermophilic Firmicutes (Bacilli and Clostridia) with additional representatives from the thermophilic Crenarchaeota and Euryarchaeota (Fukuyama et al., 2020). A modest number of mesophilic Firmicutes, Proteobacteria and methanogenic Euryarchaeota have also been isolated (Oelgeschläger and Rother, 2008; Inoue et al., 2019; Fukuyama et al., 2020). Although they are widely distributed, the ecological roles for Ni-COX in anaerobic CO cycling are uncertain. Evidence from hot springs (Brady et al., 2015) suggests that thermophilic Ni-COX contribute significantly to community metabolism if CO levels are elevated, while several additional studies have suggested that they can be exploited in various thermophilic engineered systems (Tiquia-Arashiro, 2014). In addition, metagenomic evidence supported an important role for Ni-COX in a 3-km deep Precambrian continental crust microbial community (Magnabosco et al., 2016).

Previous studies have established possible roles for mesophilic Ni-COX in anaerobic forest soils and salt marsh sediments (Conrad and Seiler, 1980; King, 2006, 2007). Conrad and Seiler (1980) documented both aerobic and anaerobic CO uptake by soil at atmospheric and sub-atmospheric concentrations. However, they also showed that pre-incubating soils anaerobically increased rates of anaerobic CO uptake while decreasing subsequent aerobic uptake rates. These results were most consistent with *aerobic* uptake by Mo-COX and *anaerobic* uptake by Ni-COX, since inhibition of the former by anaerobic pre-incubation cannot account for increased rates of anaerobic uptake.

Later work by King (2006, 2007) also documented mesophilic anaerobic CO uptake by soils at atmospheric and sub-atmospheric concentrations. For soils, the observed activity was nitrate independent and inhibited by chloroform, an Ni-CODH inhibitor (Chidthaisong and Conrad, 2000), which did not affect aerobic CO uptake (King, 2006). These results were most consistent with anaerobic CO uptake by Ni-COX. For salt marsh sediments, anaerobic activity in surface and sub-surface sediments was inhibited by nitrate and known sulfate reduction inhibitors (King, 2007). These results were consistent with anaerobic activity by sulfidogenic and acetogenic Ni-COX.

In addition to observations with soils, King (2003b,2006) showed that Mo-COX isolates (four Proteobacteria and two Actinobacteria) were unable to oxidize CO at atmospheric or sub-atmospheric concentrations under anaerobic conditions with an excess of nitrate (10 mM), although they could do so with molecular oxygen. Consequently, the known capacities of Mo-COX for nitrate-coupled CO oxidation are not consistent with *ex situ* observations of anaerobic activities in soils.

On the other hand, *Clostridium pasteurianum*, a mesophilic Ni-COX Firmicutes isolated from soil, was capable of oxidizing CO anaerobically at atmospheric concentrations as well as at 5% (Fuchs et al., 1974). Interestingly, concentrations beyond 5% inhibited uptake, which has also been observed for some soils (DePoy et al., 2020). Collectively, the previous soil and isolate results are consistent with Ni-COX rather than Mo-COX as important contributors to anaerobic CO oxidation by soils and they support recent attributions of activity in anaerobic volcanic soils to Ni-COX (e.g., DePoy et al., 2020).

Results from acetogenesis assays also support the presence and potential activity of Ni-COX in soils. Drake and collaborators have shown that acetogenesis occurred with little or no lag at mesophilic temperatures irrespective of the presence of nitrate when a range of forest and grassland soils were incubated anaerobically (Küsel and Drake, 1995; Wagner et al., 1996). Although they did not address CO transformations *per se*, acetogenic activity implies Ni-COX activity, because Ni-CODH plays a central role in acetate production (e.g., Wood et al., 1986).

Although the capacity of soils and sediments to support mesophilic anaerobic CO oxidation has received relatively little attention (e.g., Conrad and Seiler, 1980; King, 2006, 2007; DePoy et al., 2020) the available observations suggest that it might be more widespread and potentially more significant than previously realized. To address these points, anaerobic CO uptake was assayed using samples obtained from geographically dispersed sites representing a wide range of habitats. CO uptake rates were assessed under oxic and anoxic conditions with 10 ppm headspace concentrations at 25°C, and with 25% headspace concentrations under anoxic conditions at 25 and 60°C. The results revealed that Ni-COX activity occurs commonly and that it might play a role in ambient CO cycling.

## Materials and Methods

### Site Descriptions, Sample Collection and Processing

Samples for CO uptake were collected from numerous sites (see Supplementary Table 1 for site identification and location and Supplementary Tables 2–4 for pH, organic matter, collection temperature, and water contents), some of which have been described previously (King, 2003a; King et al., 2008; Weber and King, 2009; DePoy et al., 2020). The sites included geothermally heated soils (Hawai'i); boreal soils and stream sediments (Iceland); flooded agricultural (Louisiana, Japan) and wetland soils (Louisiana, Maine); hot spring sediments (Oregon); cultivated soils (Hawai'i, Louisiana, Oregon); forest soils (Hawai'i, Louisiana, Maine, Japan); arid soils (Hawai'i, Oregon); volcanic deposits (Hawai'i, Iceland, Japan); and lake sediments (Hawai'i, Louisiana). The forest site sampled in Maine was substantially similar to a site described by King (2006), while the CCRd forest sampled in Hawai'i was within about 200 m of a site previously described by King (2003a). At each site, triplicate samples [about 100-gram fresh weight (gfw)] were collected from the upper 2-cm depth interval using 70% ethanol-sterilized spatulas or trowels. Samples were transferred to zip-seal storage bags and held at ambient temperature during transport to a laboratory at Louisiana State University where they were stored at room temperature for processing. Sample analyses were typically initiated within 1 week of collection.

### Carbon Monoxide Uptake

Five-gfw samples were transferred to 60-mL serum bottles that were subsequently flushed with deoxygenated N_2_ for anoxic treatments or maintained with an air headspace for oxic treatments. For most sites, two sets of triplicates were incubated with 10 ppm CO at 25°C with oxic or anoxic headspaces, while two additional sets of triplicates were incubated with 25% CO at 25 or 60°C with anoxic headspaces. For several sites that experience cool temperatures seasonally, samples were incubated at 10°C in addition to or in lieu of incubations at 25°C; these sites included Lake Waiau (Hawai'i, LWH), Baker Swamp (Maine, BSM) and all Iceland sites. In addition, samples from Bluebonnet Swamp (BBS) and a cultivated soil on the Louisiana State University campus (LSUC) were incubated with 25% CO and temperatures from 25 to 70°C with 5 to 10°C intervals. In all cases, headspace CO concentrations were measured at intervals for samples collected with a needle and syringe for analysis by gas chromatography as described by DePoy et al. (2020). Maximum CO uptake rates were estimated from first-order rate constants or slopes of linear fits as appropriate and expressed on a dry weight basis after determining sample water contents by drying at 80°C for 48 h. For instances in which no CO uptake was observed (elevated CO only), rates were reported as zero. Apparent lag times for CO uptake were defined as the time elapsed between the initial headspace sample and the point at which a consistent decline in CO concentrations was observed. For instances in which CO uptake was not observed, lag times were considered undefined; these replicates were not included in estimates of mean lag times. For select sites, water potentials were adjusted with deionized water to levels that support microbial activity (−2 to −1 MPa). Water potentials were assessed using a WP4-T water potential meter (Decagon Devices, Pullman, WA, United States).

To assess potential inhibition of uptake by elevated CO concentrations, soil samples from BBSF and LSUC were prepared as above with anoxic headspaces and CO was added to selected final concentrations from 10 ppm to 25% CO. All treatments were prepared in triplicate. Headspace CO concentrations were analyzed as above.

An analysis of CO coupling to methanogenesis and hydrogenogenesis at 25 and 60°C was conducted using sets of triplicates from BBS prepared as above with anoxic headspaces and 25% CO. Two additional sets of triplicates were prepared with 25 mM (final concentration) bromoethanesulfonic acid (BES), an inhibitor of methanogenesis (Oremland and Capone, 1988), and with 25% CO for incubation at 25 and 60°C. CO was analyzed as described by DePoy et al. (2020). Headspace hydrogen (H_2_) concentrations up to 1% were analyzed using a Peak Laboratories (Mountain View, CA, United States) Peak Performer 2 reduced gas detector. Hydrogen concentrations >1% were analyzed using an SRI (Torrance, CA, United States) model 8610 gas chromatograph fitted with a thermal conductivity detector. Headspace methane concentrations were measured using the same instrument with a flame ionization detector and a 1-m Molecular Sieve 5A column operated with helium as a carrier gas. CO-coupled hydrogenogenesis was estimated from hydrogen concentrations based on a 1:1 hydrogen:CO stoichiometry (Diender et al., 2015). This estimate was used with the total amount of CO oxidized to calculate the relative extent of hydrogenogenesis (% of CO uptake). A similar calculation was performed for other sites where hydrogen accumulation was observed. These values would have been underestimates if hydrogen consumption occurred simultaneously with production.

### Statistical Analyses

Replicates with no observed CO uptake were assigned a zero rate, while lag times were considered indeterminant. Due to the wide range of uptake rates at 25% CO, the values were transformed prior to analysis using a log(1 + x) method. For 10 ppm CO treatments, uptake rates among sites were compared using a one-way ANOVA for each treatment without transformation. To assess differences between treatments at each site, 10 ppm uptake rates were compared using a two-way ANOVA with an interaction between site and treatment. Similarly, 25% CO uptake rates among sites were compared using a one-way ANOVA for 25 and 60°C. This was followed by a two-way ANOVA with an interaction between site and temperature. For apparent lag time comparisons, replicates with CO uptake for both 25 and 60°C were used in a paired *t*-test.

## Results

### 10 ppm Carbon Monoxide Uptake

**FIGURE 1:**
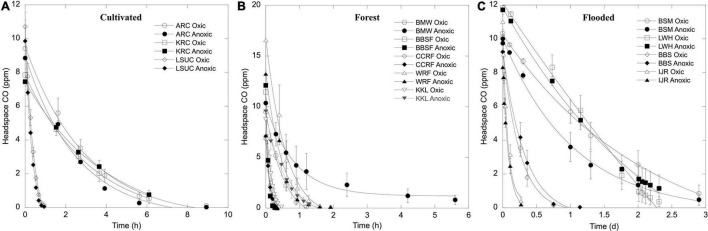
Time course of headspace CO concentrations for representative samples of **(A)** cultivated soils, **(B)** forest soils, and **(C)** flooded soils/sediments incubated with 10 ppm CO. Samples incubated with oxic headspaces are denoted with open symbols and anoxic headspaces are denoted with closed symbols. Data are means ± 1 standard error (*n* = 3).

Carbon monoxide uptake occurred with no apparent lag during aerobic and anaerobic incubations for each of the soils and sediments and uptake thresholds fell below or concentrations were reduced to atmospheric or sub-atmospheric levels at all sites (see representative results in Figure 1). Aerobic uptake rates for soils (Table 1) varied from 6 ± 3 nmol gdw^–1^ d^–1^ (MHSU) to 2042 ± 478 nmol gdw^–1^ d^–1^ (KKL-burned). Anaerobic uptake rates for soils (Table 1) varied from 5 ± 2 nmol gdw^–1^ d^–1^ (MHSU) to 1669 ± 259 nmol gdw^–1^ d^–1^ (CCRF-D18). At each of the sites, aerobic and anaerobic uptake rates were comparable (*p*_*soil*_ = 0.1035). However, CO uptake rates varied significantly among sites for oxic (*p*_*oxic*_ = 8.012e-16) and anoxic treatments (*p*_*anoxic*_ = 2.854e-11), and in general, rates decreased according to site classification as follows: forest soil > unheated volcanic soil ∼ cultivated soil > geothermally heated soil > arid soils (Table 1).

**TABLE 1:** CO uptake rates for soils, 10 ppm CO under aerobic and anaerobic conditions, and 25% CO under anaerobic conditions at 25 and 60°C.

Site	10 ppm CO uptake rate		25% CO uptake rate	
	Aerobic	Anaerobic	25°C	60°C
		
Forest soil	nmol gdw^–1^ d^–1^		μmol gdw^–1^ d^–1^	
BBSF	1184 ± 33	1224 ± 38	0	73 ± 48 (2)
BWM	282 ± 115	475 ± 21	16.8 ± 5.9	47.1 ± 47.1 (1)
MSDV	14 ± 2	6 ± 3	0	0
CCRF-D18	1600 ± 241	1669 ± 259	16.7 ± 1.8	31.7 ± 15.8 (2)
CCRF-A19	–	–	18.7 ± 4.2	113.1 ± 26.8
WRF	694 ± 146	694 ± 109	24.1 ± 3.1	133.4 ± 37.6
KKL	19 ± 3	6 ± 3	1.0 (1)	82.1 ± 4.5
KKL-burned	2043 ± 477	84 ± 38	0	46.8 ± 8.39
PGA-D18	36 ± 3	46 ± 2	0	0
PGA-D19	18 ± 5	17 ± 2	0	0
PPC	141 ± 150	1631 ± 722	0	0
CL	776 ± 70	782 ± 119	24.5 ± 1.5	154.5 ± 6.0
**Cultivated soil**				
LSUC	164 ± 3	199 ± 5	3.9 (1)	86.6 ± 7.8
KRC-J18	83 ± 11	67 ± 4	9.8 (1)	174.0 ± 34.9
KRC-J19	–	–	0.3 (1)	17.3 (1)
ARC-J18	92 ± 4.	102 ± 5	17.8 ± 9.9 (2)	114.7 ± 7.7
ARC-D18	329 ± 46	446 ± 82	31.5 ± 5.7	44.6 ± 25.8 (2)
**Geothermally heated**				
KSB-A	0^†^	0^†^	0^†^	0^†^
KSB-B	77 ± 19	93 ± 19	0	0
PGB	117 ± 43*	31 ± 19*	–	0
PGC-D18	0^†^	0^†^	0	0
PGC-A19	0^†^	19 ± 6^†^	0^†^	0^†^
**Unheated volcanic soil**				
IG-7	114 ± 20	117 ± 10	4.0 ± 2.0 (2)	31.7 ± 10.7
OY	67 ± 13	42 ± 13	0	5.8 ± 0.8
PPB	695 ± 182	433 ± 109	0	3.6 (1)
KMU	61 ± 24	22 ± 5	0	0
KHP	465 ± 353	214 ± 154	0	0
KRF-A18	210 ± 34	102 ± 5	–	82.9 ± 41.5 (2)
KRF-A19	–	–	1.7 (1)	49.2 ± 29.0 (2)
GIM	20 ± 5	24 ± 8	1.7 ± 1.2 (2)	51.9 ± 25.9 (2)
**Arid soil**				
MHSU	6 ± 3	5 ± 3	0	0
MHSP	–	–	2.5 ± 1.6 (2)	0
KRU	–	–	0	0
HCUS	23 ± 8	21 ± 7	–	–
ABPB1	–	–	12.0 (1)	0
ABPB2	–	–	0	0

*60°C; †80°C.

*All uptake rates are means ± 1 standard error (n = 3). Values in parentheses for 25% CO indicate the number of replicates that oxidized CO if fewer than 3 were active. Dashes indicate an absence of analyses. Values of zero indicate that CO uptake was not observed with a detection limit of 0.1 μmol gdw^–1^ d^–1^.*

Aerobic CO uptake rates for flooded soils and unvegetated sediments (Table 2) varied from 1 ± 0.6 nmol gdw^–1^ d^–1^ (CLR) to 964 ± 387 nmol gdw^–1^ d^–1^ (BSM). Anaerobic CO uptake rates (Table 2) varied from 3 ± 0.4 nmol gdw^–1^ d^–1^ (LSUL) to 2057 ± 426 nmol gdw^–1^ d^–1^ (BSM). In addition, aerobic and anaerobic CO uptake rates were comparable for each of the sites (*p*_*sediment*_ = 0.066) but differed among sites (*p*_*oxic*_ = 0.0027, *p*_*anoxic*_ = 1.74e-06). In general, rates for the flooded soils were substantially higher than rates for sediments.

**TABLE 2:** CO uptake rates for flooded soils and sediments, 10 ppm CO under aerobic and anaerobic conditions, and 25% CO under anaerobic conditions at 25 and 60°C.

Site	10 ppm CO uptake rate		25% CO uptake rate	
	Aerobic	Anaerobic	25°C	60°C
		
Flooded soil	nmol gdw^–1^ d^–1^		μmol gdw^–1^ d^–1^	
BBS-J18	39 ± 6	0.03 ± 0.01	157.6 ± 29.1	205.2 ± 35.2
BBS-A19	–	–	338.4 ± 100.5	45.0 ± 1.2
BSM	964 ± 387	2057 ± 426	56.0 ± 15.9	108.3 ± 55.0
IJR	41 ± 7	43 ± 6	38.5 ± 13.3	191.4 ± 47.8
CLR	1 ± 1	3 ± 1	13.0 ± 3.9	0
**Sediment**				
LWH-J18	3 ± 0.3	3 ± 0.3	19.4 ± 1.4	22.2 ± 6.9
LWH-A19	–	–	6.8 ± 0.4	14.6 ± 12.4 (2)
LSUL	2 ± 1	3 ± 0.4	18.0 ± 2.5	1.3 (1)
BLO	–	–	15.8 ± 7.5	5.0 ± 3.4 (2)
UXA	10 ± 1	13 ± 3	–	2.3 (1)

*All uptake rates are means ± 1 standard error (n = 3). Values in parentheses for 25% CO indicate the number of replicates that oxidized CO if fewer than 3 were active. Dashes indicate an absence of analyses. Values of zero indicate that CO uptake was not observed with a detection limit of 0.1 μmol gdw^–1^ d^–1^.*

### 25% Carbon Monoxide Uptake

Anaerobic CO uptake at 25% headspace concentrations occurred after lags that varied considerably. Apparent lags for soils ranged among the various sites from a few hours to >2 months (Supplementary Figure 1). For any given site, apparent lags were typically longer for soils incubated at 25°C than at 60°C (*p* = 1.41e-08), with significant differences in apparent lags among sites at both temperatures (*p* = 5.24e-05 and 9.95e-08, respectively; Supplementary Figure 1). In contrast, apparent lag times for sediments behaved more variably as a function of incubation temperature (Supplementary Figure 2). Although apparent lags differed among sites at each temperature (25°C: *p* = 1.44e-08; 60°C: *p* = 1.64e-06), the responses to incubation temperatures at individual sites varied, e.g., for BBS, apparent lags decreased at 60°C relative to 25°C while at LWH apparent lags were insensitive to incubation temperatures. Overall, apparent lag times were similar for soils and sediments at 60°C but were shorter for sediments than soils at 25°C.

Maximum uptake rates for soils incubated anaerobically with 25% CO varied considerably within and among sites (Table 1). At some sites, no uptake was observed (14 of 24 sites and 13 of 26 sites, at 25 and 60°C, respectively; Table 1). At the remaining sites, activity was observed in 1, 2, or all three replicates (Table 1). Excluding sites that did not exhibit CO uptake, rates at 25°C ranged from a low of 0.3 μmol gdw^–1^ d^–1^ (KRC-J19) to a high of 31.5 ± 5.7 μmol gdw^–1^ d^–1^ (ARC-D18). At 60°C, CO uptake rates for soils ranged from a low of 5.8 ± 0.8 μmol gdw^–1^ d^–1^ (OY) to a high of 174.0 ± 34.9 μmol gdw^–1^ d^–1^ (KRC-Jul18). CO uptake rates varied significantly among sites for 25°C (*p*_*soil*_ = 5.89e-10) and 60°C (*p*_*soil*_ = 7.68e-10) and overall were significantly higher for 60°C than for 25°C (*p*_*soil*_ = 9.723 e-08). CO uptake was not observed for geothermally heated soils obtained from Hawai'i (Table 1). In general, trends in rates among soil site types incubated at 25°C decreased as forest ∼ cultivated > arid > unheated volcanic >> geothermally heated; at 60°C, rates declined from cultivated ∼ forest > unheated volcanic.

For flooded soil and unvegetated sediment samples (Table 2), anaerobic CO uptake rates with 25% CO at 25°C ranged from 6.8 ± 0.4 μmol gdw^–1^ d^–1^ (LWH-A19) to 157.6 ± 29.1 μmol gdw^–1^ d^–1^ (BBS-J18). CO uptake rates for sediments at 60°C (Table 2) ranged from 1.3 μmol gdw^–1^ d^–1^ (LSUL) to a high of 338.4 ± 100.5 μmol gdw^–1^ d^–1^ (BBS-A19). CO uptake rates varied significantly among sites for 25°C (*p*_*sediment*_ = 6.12e-06) and 60°C (*p*_*sediment*_ = 5e-05). Uptake rates were considerably higher for flooded soils than for unvegetated sediments but in contrast with soils, CO uptake rates for 60°C were not significantly higher than for 25°C (*p*_*sediment*_ = 0.4239).

Alvord and Mickey Hot Springs sites (AHS and MHS, respectively) included temperature gradients (Supplementary Table 4). For AHS, rates for samples obtained from sites with ambient temperatures of 30 and 60°C were comparable (Table 3). However, rates were distinctly greater for samples obtained from a site at 70°C and incubated at 70°C. Rates were lowest for AHS samples obtained from 30°C and incubated at 60°C (Table 3). Rates for MHS samples obtained from sites at 25 to 35°C and incubated at 25°C were similar (Table 3), but when incubated at 60°C, rates were substantially higher for samples from sites at 35°C than from 25°C. CO uptake rates for MHS samples obtained from sites at 60°C then incubated at 60°C ranged between 38.7 ± 8.5 and 68.8 ± 6.9 μmol gdw^–1^ d^–1^ but were not distinctly different than rates for other MHS sites. No activity was observed for MHS samples collected at sites with temperatures >60°C (Table 3). Rates for two Borax Hot Springs (BHS) samples were lower than rates for other sites regardless of the site or incubation temperatures (Table 3).

**TABLE 3:** CO uptake rates (μmol gdw^–1^ d^–1^) for hot springs, 25% CO under anaerobic conditions at 25 and 60°C.

Site	25% CO uptake rate	
	25°C	60°C
MHS-J18 35	23.4 ± 11.7 (2)	97.1 ± 9.6
MHS-J18 60	–	68.8 ± 6.9
MHS-J19 25	16.2 ± 1.7	17.0 ± 8.5 (2)
MHS-J19 60	–	38.7 ± 8.5
AHS 30	40.9 ± 13.7*	14.6 ± 0.8
AHS 60	–	25.3 ± 0.8
AHS 70	–	105.5 ± 43.1^#^
BHS 46	5.4 ± 1.8†	14.4 ± 0.8
BHS 60	–	5.9 ± 3.5 (2)

*30°C; †40°C; #70°C.

*All uptake rates are means ± 1 standard error (n = 3). Values in parentheses for 25% CO indicate the number of replicates that oxidized CO if fewer than 3 were active. Dashes indicate an absence of analyses. Values of zero indicate that CO uptake was not observed with a detection limit of 0.1 μmol gdw^–1^ d^–1^.*

### Temperature Responses

**FIGURE 2:**
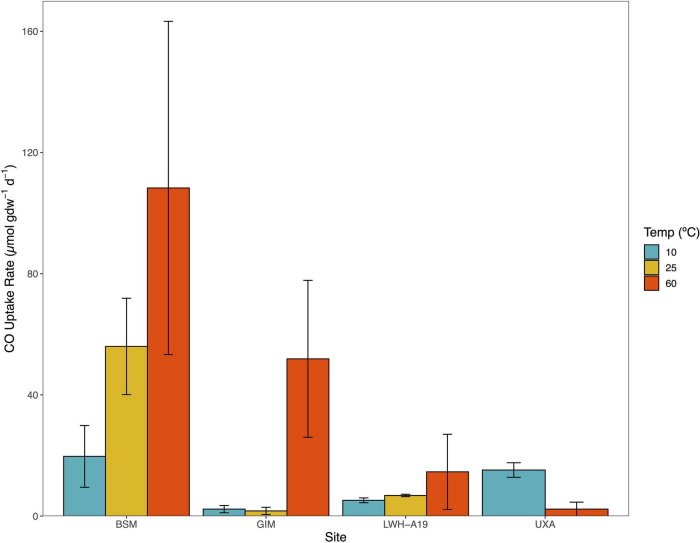
CO uptake rates (μmol gdw^–1^ d^–1^) for Baker Swamp, Maine (BSM), Grímsey Island meadow (GIM), Lake Waiau, Hawai'i April 2019 (LWH-A19), (UXA) at 10, 25, and 60°C. These sites experience extended *in situ* temperatures <25°C. CO uptake rates are means ± 1 standard error (*n* = 3).

Several sites (BSM, KRF, GIM, and LWH) were incubated anaerobically at 10°C in addition to or in lieu of incubation at 25 and 60°C. Activity at 10°C was observed for all but KRF (Figure 2) with rates ranging from 2.3 ± 1.2 μmol CO gdw^–1^ d^–1^ (GIM) to 19.7 ± 10.2 gdw^–1^ d^–1^ (BSM). These rates were comparable to values observed for many soil, flooded soil, and unvegetated sediment samples incubated at 25°C (Tables 1, 2). At BSM, rates increased distinctly from 10 to 60°C; less pronounced increases were observed for LWH (Figure 2). At GIM, there was little difference between rates at 10 and 25°C but an increase at 60°C (Figure 2). In contrast, at Uxahryggjavegur Stream (UXA) rates for 60°C incubations were distinctly lower than rates at 10°C.

**FIGURE 3:**
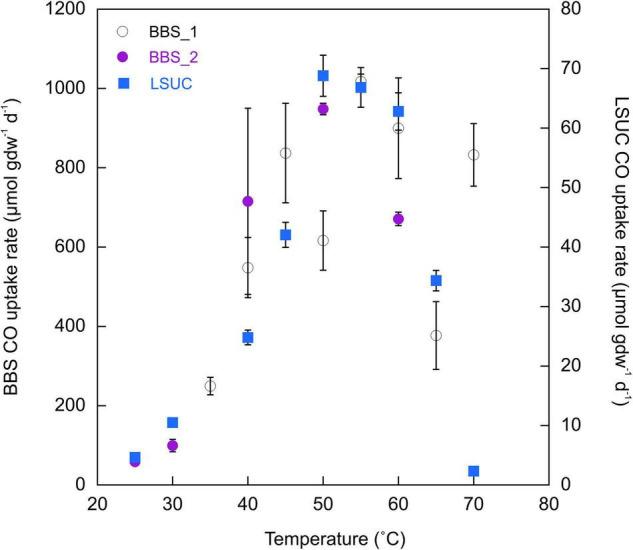
CO uptake rates (μmol gdw^–1^ d^–1^) for 25% CO at varying temperatures for Bluebonnet Swamp (BBS) (Trial 1 and Trial 2) and LSU-cultivated (LSUC). All uptake rates are means ± 1 standard error (*n* = 3).

Responses to temperatures from 25 to 70°C by LSUC samples revealed a distinct thermophilic optimum for CO uptake between 50 and 60°C with a steep decline in activity at temperatures >60°C and a more gradual decline for temperatures <50°C (Figure 3). In contrast, samples from BBS exhibited a much broader optimum from about 45 to 70°C. In addition, CO uptake rates at BBS typically exceeded rates at LSUC by ≥10-fold for given temperatures. Activation energies calculated from rates at temperatures lower than the optimum at BBS exceeded those for LSUC (133.1 kJ mol^–1^ and 96.9 kJ mol^–1^ for two BBS trials versus 83.0 kJ mol^–1^ for LSUC).

**FIGURE 4:**
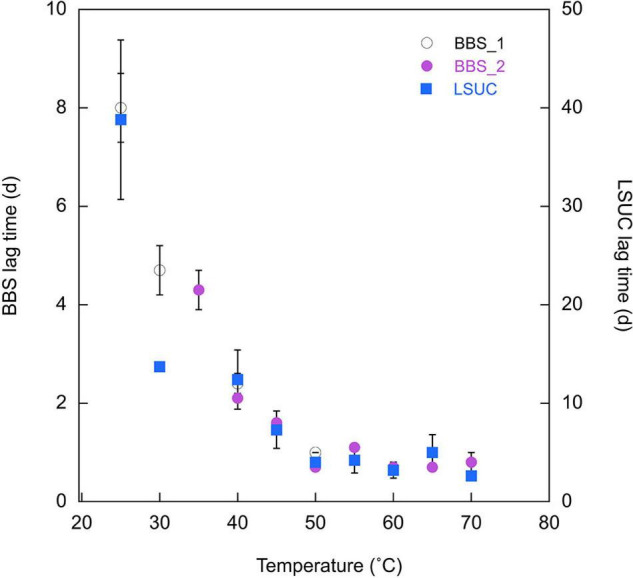
Apparent lag times (d) for 25% CO at varying temperatures for Bluebonnet Swamp (BBS) (Trial 1 and Trial 2) and LSU-cultivated (LSUC). Lag times are means ± 1 standard error (*n* = 3) with the exception of samples for which one or two replicates had undefined lag times due to absence of CO uptake; for those samples, error bars are not shown.

Apparent lag times also varied. Specifically, lag times decreased with increasing temperature between 25 and 50°C and were relatively constant at higher temperatures. Values were consistently greater for LSUC than BBS at the same incubation temperatures (Figure 4); at LSUC lag times varied from means of 2.6 to 38.8 days, while they varied from means of 0.7 to 8.0 days at BBS.

### Concentration Responses

**FIGURE 5:**
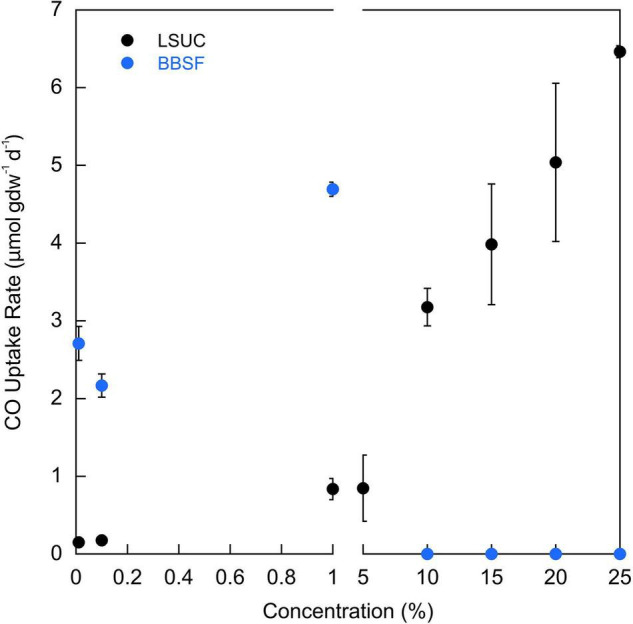
CO uptake rates (μmol gdw^–1^ d^–1^) for varying CO concentrations for LSU-cultivated (LSUC) and Bluebonnet Swamp Forest (BBSF). All uptake rates are means ± 1 standard error (*n* = 3).

LSUC soils oxidized CO anaerobically at all concentrations tested from 100 ppm to 25% when incubated at 25°C (Figure 5). Uptake rates appeared to increase and then plateau over a concentration range from 100 ppm to 5%; at higher concentrations, CO uptake rates increased linearly. In contrast, BBSF soils oxidized CO anaerobically at concentrations up to 1% but at higher concentrations uptake was completely inhibited. At CO concentrations ≤1%, uptake rates were greater for BBSF than LSUC (Figure 5).

### Hydrogenogenesis

Hydrogen accumulated in most of the samples with active anaerobic consumption of 25% CO (Table 4). For soils that produced hydrogen during incubations at 25°C, the final concentrations accounted for 7.3% (IG-7) to 67.9% (CCRF) of the added CO (Table 4), while at 60°C, they accounted for 23.0% (IG-7) to 75.5% of CO equivalents (BWM). For flooded soils and sediments incubated at 25°C, final hydrogen concentrations accounted for 4.4% (BLO) to 20.5% (BBS) of CO equivalents. At 60°C, final hydrogen concentrations accounted for 4.9% (IJR) to 71.8% (BSM) of the added CO.

**TABLE 4:** Final hydrogen concentration estimates for select soil and sediment sites.

Site	Hydrogen (% of CO equivalents)	
	25°C	60°C
**Soil**		
Baker Wood, Maine	47.4 ± 23.7	25.2 ± 25.2
CCRd Forest	67.9 ± 3.3	66.1 ± 2.0
LSU-cultivated	n.o.	49.6 ± 3.3
Bluebonnet Swamp Forest	n.o.*	30.3 ± 17.1
Amauulu	50.9 ± 3.2	33.3 ± 1.2
CL	30.1 ± 3.5	36.8 ± 1.1
IG-7	7.3	23.0 ± 11.6
**Sediment**		
Baker Swamp	n.o.	71.8 ± 2.6
Bluebonnet Swamp	20.5 ± 4.9	39.1 ± 5.4
Ibaraki Rice	8.4 ± 6.1	4.9 ± 6.6
Borax Lake	4.4 ± 2.3	16.1 ± 1.6

Concentrations are expressed as percent (%) of CO equivalents. An asterisk (*) indicates no CO uptake was observed for the samples; n.o., indicates hydrogen production not observed during the course of the assays. Values are means ± 1 standard error (n = 3) with the exception of IG-7 at 25°C for which only one replicate produced hydrogen.

During an analysis of BBS samples, methane accounted for 7.4 ± 2.5% and 0.1 ± 0.02% of CO equivalents at 25 and 60°C, respectively, using a 4:1 CO:CH_4_ stoichiometry (Diender et al., 2015). In the presence of 25 mM BES (final concentration) as a methanogenesis inhibitor, methane accounted for 0.2 ± 0.1% and 0.1 ± 0.01 of CO equivalents at 25 and 60°C, respectively. In the absence of BES, hydrogen accounted for 22.3 ± 13.5% and 66.7 ± 0.9% of CO equivalents at 25 and 60°C, respectively. In the presence of BES, hydrogen accounted for 21.5 ± 13.8% and 66.3 ± 0.2 of CO equivalents, respectively.

## Discussion

### Uptake of 10 ppm Carbon Monoxide

In this study, the capacity for anaerobic and aerobic CO uptake at 10 ppm was assessed for a wide range of soils and sediments. Although higher than atmospheric levels, the initial headspace concentrations of samples at all sites were reduced to sub-atmospheric levels or thresholds with no apparent lags and at rates that were comparable for oxic and anoxic incubations (Figure 1 and Tables 1, 2).

Anaerobic CO uptake could be attributed to Ni-COX based on outcomes of previously published studies. Fuchs et al. (1974) showed that a mesophilic Ni-COX soil isolate, *Clostridium pasteurianum*, could oxidize CO at atmospheric concentrations as well as concentrations up to 5%. Numerous other Ni-COX isolates have been enriched with CO concentrations up to 100% though their ability to use atmospheric CO is uncertain.

In contrast, several Mo-COX isolates oxidized CO anaerobically with nitrate, but none were able to do so at atmospheric concentrations even though they could all do so aerobically (King, 2003b,^2006^). The latter observation suggested that atmospheric CO oxidation was not simply a property of the Mo-CODH, but that it was constrained by growth conditions, e.g., anaerobiosis. More importantly, the isolate evidence did not support attribution of anaerobic CO uptake by soils to Mo-COX.

Responses of soils to various treatments were also inconsistent with Mo-COX activity under anaerobic conditions. Conrad and Seiler (1980) reported that anaerobic pre-incubation inhibited subsequent aerobic CO uptake while anaerobic uptake was stimulated. The anaerobic activity is best attributed to Ni-COX not Mo-COX. King (2006) subsequently showed that anaerobic CO uptake by forest soils was nitrate independent but inhibited by chloroform, a known Ni-CODH inhibitor that did not affect aerobic uptake (Chidthaisong and Conrad, 2000). In addition, all of the soils in these studies oxidized CO at atmospheric and sub-atmospheric concentrations, a capacity that has not been documented for Mo-COX under anaerobic conditions.

Collectively results from past studies and the present work support several possibilities. First, they show that Ni-COX activity might be initiated under anaerobic conditions on the same time scales as those for Mo-COX irrespective of sample type or source. This may indicate that Ni-COX populations are routinely active in anoxic microzones or that Ni-COX populations are activated very rapidly (<<1 h) after anaerobic conditions are established, a result comparable to that reported by Bartholomew and Alexander (1979). Second, the results indicate that during periods of anaerobiosis, Ni-COX might use the atmosphere as either a primary CO source or as a supplement to CO produced endogenously (e.g., King and Crosby, 2002). Third, comparable rates for aerobic and anaerobic CO uptake in most samples (Tables 1, 2) suggest that Ni-COX could play potentially important and previously unrecognized roles in soil-atmosphere CO exchanges. The magnitude of these contributions warrants additional attention.

The geographically widespread distribution of anaerobic CO uptake at 10 ppm in a broad range of soils and sediments (Tables 1–3) also indicates that Ni-COX could extend well beyond thermophilic systems that have been the focus of recent assays (Techtmann et al., 2009; Kochetkova et al., 2011; Brady et al., 2015; Yoneda et al., 2015; Omae et al., 2021). Indeed, this study suggests that Ni-COX and their activities might be considered ubiquitous, likely involving numerous uncharacterized mesophiles. Although the roles and significance of Ni-COX remain to be determined, assessing their diversity and functions represent opportunities to develop novel insights about CO cycling.

### Uptake of 25% Carbon Monoxide

Anaerobic uptake of 25% CO cannot plausibly be attributed to Mo-COX for two principle reasons. First, Mo-COX are inhibited by high CO concentrations when respiring nitrate; thus, they could not have oxidized CO under the incubation conditions used in this study (Frunzke and Meyer, 1990; King, 2006). Second, the amount of CO added, about 700 μmol, vastly exceeded the amount of nitrate typically available in soils; thus, even if anaerobic respiration was possible, the only known alternate electron acceptor for Mo-COX could have supported no more than a negligible fraction of the observed CO uptake. In addition, hydrogenogenesis was observed in response to CO addition; Mo-COX possess no known mechanism that could account for the water-gas shift reaction.

In contrast, Ni-COX traits readily explain the results. First, numerous Ni-COX have been isolated from soils and sediments with elevated CO concentrations and incubation conditions similar to those used in this study (e.g., Fuchs et al., 1974; Parshina et al., 2005; Slepova et al., 2006; Ahmed et al., 2010; Bertsch and Müller, 2015; Geelhoed et al., 2016). These isolates include metabolically diverse mesophiles and many thermophiles that can account for results at 25 and 60°C. In addition, only Ni-COX metabolize CO *via* hydrogenogenesis; therefore, they best explain CO-coupled hydrogen production, which was measured routinely.

Thus, the collective anaerobic CO uptake activity observed in this study strongly suggests that both mesophilic and thermophilic Ni-COX are widely distributed geographically (Tables 1–3). Anaerobic activity at mesophilic temperatures occurred in soils and sediments but was most consistent in the latter. Anaerobic CO uptake was even observed at psychrotrophic temperatures (10°C) for four sites that experienced low temperatures *in situ* (Figure 2). At present, the taxa responsible for mesophilic (10 ppm and 25% CO) and psychrotrophic (25% CO) uptake are uncertain, as is the extent to which the same taxa might use both near ambient and elevated CO concentrations.

Although results from this study provide evidence for the ubiquity of mesophilic Ni-COX, thermophilic Ni-COX appear equally widespread among mesothermal systems and they may prove even more abundant with greater potential activity. While previous studies have documented heterotrophic thermophiles in various soils and sediments (Marchant, 2002; Marchant et al., 2008; Perfumo and Marchant, 2010; Zeigler, 2014; Cockell et al., 2015) and thermophilic Ni-COX in geothermally heated systems (Slepova et al., 2006; Pakshirajan and Mal, 2013; Esquivel-Elizondo et al., 2017), this study shows that processes that have been invoked to explain the distribution of the former (Marchant et al., 2008; Perfumo and Marchant, 2010; Zeigler, 2014) might apply to the latter. Specifically, Ni-COX might be transported through the atmosphere as has been proposed for *Geobacillus* (Marchant et al., 2008). However, since some Ni-COX are not spore-formers (e.g., *Geobacter*) additional information on Ni-COX populations will be necessary to evaluate the hypothesis.

Regardless of their origin, soil and sediment microbial seed banks are comprised of thermophilic Ni-COX that respond to anoxia, elevated temperatures, and high CO concentrations. Several lines of evidence suggest that these thermophiles have a greater capacity to use elevated CO than do soil mesophiles. For instance, maximum uptake rates for soils incubated at 60°C were higher than for those incubated at 25°C for fifteen of twenty samples with activity at one or both temperatures (Table 1). This could reflect larger thermophilic than mesophilic Ni-COX communities. That in turn raises the possibility that aerobic mesothermal soils are more favorable for the maintenance of thermophilic than mesophilic Ni-COX populations. This counterintuitive outcome might occur if periodic oscillations between oxic and anoxic conditions at ambient temperatures subject mesophilic Ni-COX populations to greater mortality rates than those experienced by thermophilic Ni-COX that require rarely encountered high temperatures and anoxia for activation, and that consequently experience lower mortality. This proposal is consistent with explanations offered to account for anomalously high populations of thermophilic *Geobacillus* in soils (Perfumo and Marchant, 2010).

Shorter apparent lag times for soils incubated at 60 versus 25°C also suggest a greater capacity for thermophilic Ni-COX (Supplementary Figure 1). With just a few exceptions, apparent lag times were distinctly shorter at 60°C, often by a factor of 5–10. In this study, apparent lag times likely reflect in part the sizes of populations that depleted the added CO. Larger active Ni-COX populations that adapted to and grew more quickly with CO could have resulted in more rapidly detectable CO depletion.

Nonetheless, it is noteworthy that maximum anaerobic CO uptake rates at 25°C were greater than or comparable to rates at 60°C for eight of nine flooded soil and sediment sites (Table 2). This could indicate that mesophilic and thermophilic Ni-COX population sizes are more similar in systems where anoxia is generally prevalent, e.g., flooded soils. Routine anoxia at ambient temperatures could even favor greater populations of mesophilic Ni-COX and thus account for greater activity at 25°C.

Comparisons of apparent lag times at 25 and 60°C for flooded soils and sediments (Supplementary Figure 2) support this interpretation. At five of eight sites with paired data, apparent lag times at 25°C were less than or comparable to those at 60°C. In addition, apparent lag times at 25°C were typically shorter for sediment samples than for soils, while the reverse was true for 60°C apparent lag times (Supplementary Figures 1, 2).

Collectively, these observations suggest that different variables govern soil Ni-COX populations and their activity relative to those in flooded soils and sediments. However, a temperature response assay using a cultivated soil (LSUC) revealed a narrow thermophilic optimum of 50–60°C for CO uptake, while a somewhat broader yet thermotolerant to thermophilic optimum between 40 and 70°C was observed for Bluebonnet Swamp (Figure 2). Thus, generalizations about the distribution, activity, and controls of specific Ni-COX groups must remain constrained until data from additional sites are available.

Data for geothermally heated soils and hot spring sediments illustrate the need for further analysis. Although numerous thermophilic Ni-COX have been described (Sokolova et al., 2001, 2002, 2004, 2005; Techtmann et al., 2009; Novikov et al., 2011; Slepova et al., 2006; Alves et al., 2013; Kochetkova et al., 2020), CO uptake at 25% concentrations was not observed for any of the geothermally heated soil samples (Table 1). In contrast, CO was consumed by five of seven hot spring sediment samples obtained from sites with temperatures of 60–86°C (Table 3). The two exceptions were 69 and 86°C sediment from Mickey Hot Springs incubated at 70 and 86°C, respectively, for which no uptake was observed. These observations suggest that hot spring sediments are more permissive for Ni-COX than geothermally heated soils but the underlying mechanisms remain unclear. Low pH for one of the geothermal soils (pH = 2.6, KSB-A) could constrain activity, but that does not account for the other sites for which pH values were more permissive. Differences in aeration might be determinative but they have not yet been evaluated.

Inhibition of anaerobic uptake by elevated CO concentrations offers an explanation for at least some samples that were active with 10 ppm CO but not 25% concentrations. CO inhibition of Mo-COX has been reported for concentrations >1000 ppm (King, 2003b) and variable responses have also been reported for Ni-COX (Lorowitz and Bryant, 1984; Setubal et al., 2009). Although many Ni-COX isolates have been obtained using CO concentrations >50% in enrichments (Sokolova et al., 2002, 2004, 2005; Techtmann et al., 2009; Novikov et al., 2011; Kochetkova et al., 2020) others have been inhibited by concentrations <20% and even concentrations as low as 0.5% (Lorowitz and Bryant, 1984; Setubal et al., 2009). Inhibition has also been described for volcanic soils (DePoy et al., 2020). In this study BBSF soils were inhibited by CO concentrations >1%, while no inhibition was noted for LSUC soils (Figure 5). These and prior results (DePoy et al., 2020) suggest that inhibition might have limited the responses of some soils but not others. Variability in inhibition could result from differences among soils or soil replicates in the Ni-COX seed bank along with stochasticity in the activation of dormant populations. Interestingly, inhibition appears to have been more pronounced in soils than in sediments and for incubations at 25°C (e.g., Tables 1, 2). The possibility that CO uptake by Ni-COX might be inhibited by CO concentrations >1–5% needs further attention to help establish methods suitable for comparative analyses.

### Hydrogenogenesis

The extent of CO-coupled hydrogen production (referred to here as hydrogenogenesis) during anaerobic incubations of soils and sediments offers an additional perspective on Ni-COX activity. Much of what is known about hydrogenogenesis has been derived from thermophilic isolates with relatively little focus on natural systems (Sokolova et al., 2001, 2002, 2004, 2005, 2009; Slepova et al., 2006). Limited exceptions include analyses of thermophilic bioreactors and hot spring sediments (Kochetkova et al., 2011; Pakshirajan and Mal, 2013; Brady et al., 2015). While results from these studies include observations relevant for understanding the ecology of hydrogenogenesis, they provide little general information on distribution, activity, temperature responses, or responses to other variables.

Results from this study provide new insights. First, observations of anaerobic mesophilic and thermophilic activity in a wide range of soils and sediments are consistent with a geographically ubiquitous distribution for hydrogenogenesis (Table 4). Although several mesophilic CO-oxidizing hydrogenogens have been isolated from anaerobic digestors and freshwater sources (Lorowitz and Bryant, 1984; Maness and Weaver, 2002; Setubal et al., 2009; Wawrousek et al., 2014), the diversity of these taxa is unknown but likely substantial. In support of this proposition, Inoue et al. (2019) have recently identified >70 candidate mesophilic CO-oxidizing hydrogenogens based on genomic analyses. Additional analyses are warranted.

Second, results from both soils and sediments suggest that the relative significance of CO-coupled hydrogenogenesis might depend on available CO concentrations, since hydrogen production was observed during incubations with 25% CO but not with 10 ppm levels. While hydrogen production from 10 ppm CO might have been obscured by simultaneous hydrogen uptake, it is also possible that high CO concentrations are required to induce activity since the free energy yield (ΔG^0^) for hydrogenogenesis is relatively low compared to that for other oxidation pathways (Diender et al., 2015). Additional analyses of responses to CO concentrations will help clarify this point.

Finally, estimates of the relative significance of CO-coupled hydrogenogenesis are comparable for soils and sediments and for mesophilic and thermophilic incubations (Table 4). Though the outcomes vary significantly, hydrogenogenesis also accounts for a substantial fraction of anaerobic CO metabolism (>30%) at most sites (Table 4). These novel results suggest that CO-oxidizing hydrogenogens are important members of Ni-COX communities that warrant further attention.

## Conclusion

Results from this study offer novel insights about a process previously thought to be associated primarily with hot springs and bioreactors. They reveal a widespread and unsuspected anaerobic capacity to oxidize CO at low concentrations, including atmospheric levels, which suggests that Ni-COX might contribute to CO dynamics *in situ*, including soil-atmosphere CO exchanges. Though not relevant for understanding CO uptake under *in situ* conditions, activity at 25% concentrations indicates that psychrotrophic, mesophilic, and thermophilic Ni-COX carboxydotrophs all occur commonly in soils and sediments with the former potentially active at low CO concentrations. In addition, hydrogenogenic CO metabolism occurred at 25 and 60°C, in some cases accounting for a large fraction of CO uptake. Collectively these observations demonstrate that Ni-COX are an ecologically versatile functional group about which much remains to be learned.

## Data Availability Statement

The original contributions presented in the study are included in the article/Supplementary Material, further inquiries can be directed to the corresponding author.

## Author Contributions

AD and GK contributed equally to experimental design, sample collection and processing, sample analysis, data analysis, and authorship. Both authors contributed to the article and approved the submitted version.

## Conflict of Interest

The authors declare that the research was conducted in the absence of any commercial or financial relationships that could be construed as a potential conflict of interest.

## Publisher’s Note

All claims expressed in this article are solely those of the authors and do not necessarily represent those of their affiliated organizations, or those of the publisher, the editors and the reviewers. Any product that may be evaluated in this article, or claim that may be made by its manufacturer, is not guaranteed or endorsed by the publisher.

## References

[B1] Ahmed N. Bruant G. Lévesque M.-J. Peter C. Guiot S. R. Masson L. (2010). Genomic analysis of carbon monoxide utilization and butanol production by *Clostridium carboxidivorans* strain P7T. *PLoS One* 5:e13033. 10.1371/journal.pone.0013033 20885952 PMC2946384

[B2] Alves J. I. van Gelder A. H. Alves M. M. Sousa D. Z. Plugge C. M. (2013). *Moorella stamsii* sp. nov., a new anaerobic thermophilic hydrogenogenic carboxydotroph isolated from digester sludge. *Int. J. Syst. Evol. Microbiol.* 63 4072–4076. 10.1099/ijs.0.050369-0 23749275

[B3] Bartholomew G. W. Alexander M. (1979). Microbial metabolism of carbon monoxide in culture and in soil. *Appl. Environ. Microbiol.* 37 932–937. 10.1128/aem.37.5.932-937.1979 485139 PMC243327

[B4] Bertsch J. Müller V. (2015). CO metabolism in the acetogen *Acetobacterium woodii*. *Appl. Environ. Microbiol.* 81, 5949–5956. 10.1128/AEM.01772-15 26092462 PMC4551271

[B5] Brady A. L. Sharp C. E. Grasby S. E. Dunfield P. F. (2015). Anaerobic carboxydotrophic bacteria in geothermal springs identified using stable isotope probing. *Front. Microbiol.* 6:897. 10.3389/fmicb.2015.00897 26388850 PMC4555085

[B6] Chidthaisong A. Conrad R. (2000). Turnover of glucose and acetate coupled to reduction of nitrate, ferric iron and sulfate and to methanogenesis in anoxic rice field soil. *FEMS Microbiol. Ecol.* 31 73–86. 10.1016/S0168-6496(99)00083-5 10620721

[B7] Cockell C. S. Cousins C. Wilkinson P. T. Olsson-K. Rozitis B. (2015). Are thermophilic microorganisms active in cold environments? *Int. J. Astrobiol.* 14 457–463.

[B8] Conrad R. Seiler W. (1980). Role of microorganisms in the consumption and production of atmospheric carbon monoxide by soil. *Appl. Environ. Microbiol.* 40, 437–445. 10.1128/aem.40.3.437-445.1980 16345624 PMC291601

[B9] Cordero P. R. F. Bayly K. Man Leung P. Huang C. Islam Z. F. Schittenhelm R. B. (2019). Atmospheric carbon monoxide oxidation is a widespread mechanism supporting microbial survival. *ISME J.* 13 2868–2881. 10.1038/s41396-019-0479-8 31358912 PMC6794299

[B10] DePoy A. N. King G. M. Ohta H. (2020). Anaerobic carbon monoxide uptake by microbial communities in volcanic deposits at different stages of successional development on O-yama volcano, Miyake-jima, Japan. *Microorganisms* 9:12. 10.3390/microorganisms9010012 33375160 PMC7822213

[B11] Diender M. Stams A. J. M. Sousa D. Z. (2015). Pathways and bioenergetics of anaerobic carbon monoxide fermentation. *Front. Microbiol.* 6:1275. 10.3389/fmicb.2015.01275 26635746 PMC4652020

[B12] Esquivel-Elizondo S. Delgado A. G. Krajmalnik-Brown R. (2017). Evolution of microbial communities growing with carbon monoxide, hydrogen, and carbon dioxide. *FEMS Microbiol. Ecol.* 93 1–12. 10.1093/femsec/fix076 28575426

[B13] Frunzke K. Meyer O. (1990). Nitrate respiration, denitrification, and utilization of nitrogen sources by aerobic carbon monoxide-oxidizing bacteria. *Arch. Microbiol.* 154 168–174.

[B14] Fuchs G. Schnitker U. Thauer R. K. (1974). Carbon monoxide oxidation by growing cultures of *Clostridium pasteurianum*. *Eur. J. Biochem.* 49 111–115. 10.1111/j.1432-1033.1974.tb03816.x 4459138

[B15] Fukuyama Y. Inoue M. Omae K. Yoshida T. Sako Y. (2020). Anaerobic and hydrogenogenic carbon monoxide-oxidizing prokaryotes: versatile microbial conversionof a toxic gas into an available energy. *Adv. Appl. Microbiol.* 110 99–148. 10.1016/bs.aambs.2019.12.001 32386607

[B16] Geelhoed J. S. Henstra A. M. Stams A. J. M. (2016). Carboxydotrophic growth of *Geobacter sulfurreducens*. *Appl. Microbiol. Biotechnol.* 100, 997–1007. 10.1007/s00253-015-7033-z 26481622 PMC4703632

[B17] Inoue M. Nakamoto I. Omae K. Oguro T. Ogata H. Yoshida T. (2019). Structural and phylogenetic diversity of anaerobic carbon-monoxide dehydrogenases. *Front. Microbiol.* 9:3353. 10.3389/fmicb.2018.03353 30705673 PMC6344411

[B18] King G. M. (1999). Characteristics and significance of atmospheric carbon monoxide consumption by soils. *Chemosph. Glob. Change Sci.* 1 53–63. 10.1016/S1465-9972(99)00021-5

[B19] King G. M. (2003a). Molecular and culture-based analyses of aerobic carbon monoxide oxidizer diversity. *Appl. Environ. Microbiol.* 69 7257–7265. 10.1128/AEM.69.12.7257-7265.2003 14660374 PMC309980

[B20] King G. M. (2003b). Contributions of atmospheric CO and hydrogen uptake to microbial dynamics on recent Hawaiian volcanic deposits. *Appl. Environ. Microbiol.* 69 4067–4075. 10.1128/AEM.69.7.4067-4075.2003 12839783 PMC165208

[B21] King G. M. (2006). Nitrate-dependent anaerobic carbon monoxide oxidation by aerobic CO-oxidizing bacteria. *FEMS Microbiol. Ecol.* 56 1–7. 10.1111/j.1574-6941.2006.00065.x 16542399

[B22] King G. M. (2007). Microbial carbon monoxide consumption in salt marsh sediments. *FEMS Microbiol. Ecol.* 59 2–9. 10.1111/j.1574-6941.2006.00215.x 17059484

[B23] King G. M. Crosby H. (2002). Impacts of plant roots on soil CO cycling and soil-atmosphere CO exchange. *Glob. Change Biol.* 8 1085–1093. 10.1046/j.1365-2486.2002.00545.x

[B24] King G. M. Weber C. F. (2007). Distribution, diversity and ecology of aerobic CO-oxidizing bacteria. *Nat. Rev. Microbiol.* 5 107–118. 10.1038/nrmicro1595 17224920

[B25] King G. M. Weber C. F. Nanba K. Sato Y. Ohta H. (2008). Atmospheric CO and hydrogen uptake and CO oxidizer phylogeny for Miyake-jima, Japan volcanic deposits. *Microbes Environ.* 23 299–305. 10.1264/jsme2.ME08528 21558722

[B26] Kochetkova T. V. Mardanov A. V. Sokolova T. G. Bonch-Osmolovskaya E. A. Kublanov I. V. Kevbrin V. V. (2020). The first crenarchaeon capable of growth by anaerobic carbon monoxide oxidation coupled with H2 production. *Syst. Appl. Microbiol.* 43:126064. 10.1016/j.syapm.2020.126064 32044151

[B27] Kochetkova T. V. Rusanov I. I. Pimenov N. V. Kolganova T. V. Lebedinsky A. V. Bonch-Osmolovskaya E. A. (2011). Anaerobic transformation of carbon monoxide by microbial communities of Kamchatka hot springs. *Extremophiles* 15 319–325. 10.1007/s00792-011-0362-7 21387195

[B28] Küsel K. Drake H. L. (1995). Effects of environmental parameters on the formation and turnover of acetate by forest soils. *Appl. Environ. Microbiol.* 61 3667–3675. 10.1128/aem.61.10.3667-3675.1995 16535147 PMC1388709

[B29] Lorowitz W. H. Bryant M. P. (1984). Peptostreptococcus productus strain that grows rapidly with CO as the energy source. *Appl. Environ. Microbiol.* 47 961–964. 10.1128/aem.47.5.961-964.1984 6430231 PMC240027

[B30] Magnabosco C. Ryan K. Lau M. C. Y. Kuloyo O. Lollar B. S. Kieft T. L. (2016). A metagenomic window into carbon metabolism at 3 km depth in Precambrian continental crust. *ISME J.* 10 730–741. 10.1038/ismej.2015.150 26325359 PMC4817676

[B31] Maness P. C. Weaver P. F. (2002). Hydrogen production from a carbon-monoxide oxidation pathway in *Rubrivivax gelatinosus*. *Int. J. Hydrogen Energy* 27 1407–1411. 10.1016/S0360-3199(02)00107-6

[B32] Marchant R. (2002). What are high-temperature bacteria doing in cold environments? *Trends Microbiol.* 10 120–121. 10.1016/s0966-842x(02)02311-9 11864820

[B33] Marchant R. Franzetti A. Pavlostathis S. G. Tas D. O. Erdbrügger I. Ünyayar A. (2008). Thermophilic bacteria in cool temperate soils: are they metabolically active or continually added by global atmospheric transport? *Appl. Microbiol. Biotechnol.* 78 841–852. 10.1007/s00253-008-1372-y 18256821

[B34] Novikov A. A. Sokolova T. G. Lebedinsky A. V. Kolganova T. V. Bonch-Osmolovskaya E. A. (2011). *Carboxydothermus islandicus* sp. nov., a thermophilic, hydrogenogenic, carboxydotrophic bacterium isolated from a hot spring. *Int. J. Syst. Evol. Microbiol.* 61 2532–2537. 10.1099/ijs.0.030288-0 21131500

[B35] Oelgeschläger E. Rother M. (2008). Carbon monoxide-dependent energy metabolism in anaerobic bacteria and archaea. *Arch. Microbiol.* 190 257–269. 10.1007/s00203-008-0382-6 18575848

[B36] Omae K. Oguro T. Inoue M. Fukuyama Y. Yoshida T. Sako Y. (2021). Diversity analysis of thermophilic hydrogenogenic carboxydotrophs by carbon monoxide dehydrogenase amplicon sequencing using new primers. *Extremophiles* 25 61–76. 10.1007/s00792-020-01211-y 33415441 PMC7811984

[B37] Oremland R. S. Capone D. G. (1988). “Use of ‘specific’ inhibitors in biogeochemistry and microbial ecology,” in *Advances in Microbial Ecology*, ed. Marshall K. C. (New York: Plenum Press), 285–383. 10.1007/978-1-4684-5409-3_8

[B38] Pakshirajan K. Mal J. (2013). Biohydrogen production using native carbon monoxide converting anaerobic microbial consortium predominantly *Petrobacter* sp. *Int. J. Hydrogen Energy* 38 16020–16028. 10.1016/j.ijhydene.2013.09.129

[B39] Parshina S. N. Sipma J. Nakashimada Y. Henstra A. M. Smidt H. Lysenko A. M. (2005). *Desulfotomaculum carboxydivorans* sp. nov., a novel sulfate-reducing bacterium capable of growth at 100% CO. *Int. J. Syst. Evol. Microbiol*. 55, 2159–2165. 10.1099/ijs.0.63780-0 16166725

[B40] Perfumo A. Marchant R. (2010). Global transport of thermophilic bacteria in atmospheric dust. *Environ. Microbiol. Rep.* 2 333–339. 10.1111/j.1758-2229.2010.00143.x 23766086

[B41] Ragsdale S. W. (2004). Life with carbon monoxide. *Crit. Rev. Biochem. Mol. Biol.* 39 165–195. 10.1080/10409230490496577 15596550

[B42] Setubal J. C. dos Santos P. Goldman B. S. Ertesvag H. Espin G. Rubio L. M. (2009). Genome sequence of *Azotobacter vinelandii*, an obligate aerobe specialized to support diverse anaerobic metabolic processes. *J. Bacteriol.* 191 4534–4545. 10.1128/JB.00504-09 19429624 PMC2704721

[B43] Slepova T. V. Sokolova T. G. Lysenko A. M. Tourova T. P. Kolganova T. V. Kamzolkina O. V. (2006). *Carboxydocella sporoproducens* sp. nov., a novel anaerobic CO-utilizing/H_2_-producing thermophilic bacterium from a Kamchatka hot spring. *Int. J. Syst. Evol. Microbiol.* 56 797–800. 10.1099/ijs.0.63961-0 16585697

[B44] Sokolova T. G. González J. M. Kostrikina N. A. Chernyh N. A. Slepova T. V. Bonch-Osmolovskaya E. A. (2004). *Thermosinus carboxydivorans* gen. nov., sp. nov., a new anaerobic, thermophilic, carbon-monoxide-oxidizing, hydrogenogenic bacterium from a hot pool of Yellowstone National Park. *Int. J. Syst. Evol. Microbiol.* 54 2353–2359. 10.1099/ijs.0.63186-0 15545483

[B45] Sokolova T. G. Henstra A. M. Sipma J. Parshina S. N. Stams A. J. M. Lebedinsky A. V. (2009). Diversity and ecophysiological features of thermophilic carboxydotrophic anaerobes. *FEMS Microbiol. Ecol.* 68 131–141. 10.1111/j.1574-6941.2009.00663.x 19573196

[B46] Sokolova T. G. Kostrikina N. A. Chernyh N. A. Kolganova T. V. Tourova T. P. Bonch-Osmolovskaya E. A. (2005). *Thermincola carboxydiphila* gen. nov., sp. nov., a novel anaerobic, carboxydotrophic, hydrogenogenic bacterium from a hot spring of the Kae Baikal area. *Int. J. Syst. Evol. Microbiol.* 55 2069–2073. 10.1099/ijs.0.63299-0 16166711

[B47] Sokolova T. G. Kostrikina N. A. Chernyh N. A. Tourova T. P. Kolganova T. V. Bonch-Osmolovskaya E. A. (2002). *Carboxydocella thermautotrophica* gen. nov., sp. nov., a novel anaerobic, CO-utilizing thermophile from a Kamchatkan hot spring. *Int. J. Syst. Evol. Microbiol.* 52 1961–1967. 10.1099/ijs.0.02173-0 12508854

[B48] Sokolova T. González J. Kostrikina N. Chernyh N. Tourova T. Kato C. (2001). *Carboxydobrachium pacificum* gen. nov., sp. nov., a new anaerobic, thermophilic, CO-utilizing marine bacterium from Okinawa Trough. *Int. J. Syst. Evol. Microbiol.* 51 141–149. 10.1099/00207713-51-1-141 11211251

[B49] Techtmann S. M. Colman A. S. Robb F. T. (2009). That which does not kill us only makes us stronger: the role of carbon monoxide in thermophilic microbial consortia: minireview. *Environ. Microbiol.* 11 1027–1037. 10.1111/j.1462-2920.2009.01865.x 19239487

[B50] Techtmann S. M. Colman A. S. Murphy M. B. Schackwitz W. S. Goodwin L. A. Robb F. T. (2011). Regulation of multiple carbon monoxide consumption pathways in anaerobic bacteria. *Front. Microbiol.* 2:147. 10.3389/fmicb.2011.00147 21808633 PMC3135865

[B51] Tiquia-Arashiro S. M. (2014). *Thermophilic Carboxydotrophs and their Applications in Biotechnology.* New York: Springer.

[B52] Wagner C. Grießhammer A. Drake H. L. (1996). Acetogenic capacities and the anaerobic turnover of carbon in a Kansas prairie soil. *Appl. Environ. Microbiol.* 62 494–500. 10.1128/aem.62.2.494-500.1996 16535237 PMC1388775

[B53] Wawrousek K. Noble S. Korlach J. Chen J. Eckert C. Yu J. (2014). Genome annotation provides insight into carbon monoxide and hydrogen metabolism in *Rubrivivax gelatinosus*. *PLoS One* 9:e114551. 10.1371/journal.pone.0114551 25479613 PMC4257681

[B54] Weber C. F. King G. M. (2009). Water stress impacts on bacterial carbon monoxide oxidation on recent volcanic deposits. *ISME J.* 3 1325–1334. 10.1038/ismej.2009.70 19641536

[B55] Wood H. G. Ragsdale S. W. Pezacka E. (1986). The acetyl-CoA pathway: a newly discovered pathway of autotrophic growth. *Trends Biochem. Sci.* 11 14–18. 10.1016/0968-0004(86)90223-9

[B56] Yoneda Y. Kano S. I. Yoshida T. Ikeda E. Fukuyama Y. Omae K. (2015). Detection of anaerobic carbon monoxide-oxidizing thermophiles in hydrothermal environments. *FEMS Microbiol. Ecol.* 91:fiv093. 10.1093/femsec/fiv093 26223231

[B57] Zeigler D. R. (2014). The Geobacillus paradox: why is a thermophilic bacterial genus so prevalent on a mesophilic planet? *Microbiology* 159 1–11. 10.1099/mic.0.071696-0 24085838

