# Supplementary material for: Putative Nickel-Dependent Anaerobic Carbon Monoxide Uptake Occurs Commonly in Soils and Sediments at Ambient Temperature and Might Contribute to Atmospheric and Sub-Atmospheric Carbon Monoxide Uptake During Anoxic Conditions
Source: Front Microbiol. 2022 Mar 24;13:736189. doi: 10.3389/fmicb.2022.736189 (PMC8987735; doi:10.3389/fmicb.2022.736189)
Supplement: Supplementary file 1 [file Data_Sheet_1.PDF]

Figure S1. Apparent lag times (d) for soil sites. Lag times are shown at 25 ºC (A) and at 60 ºC (B). Median values are indicated by solid bars.

Figure S1A.

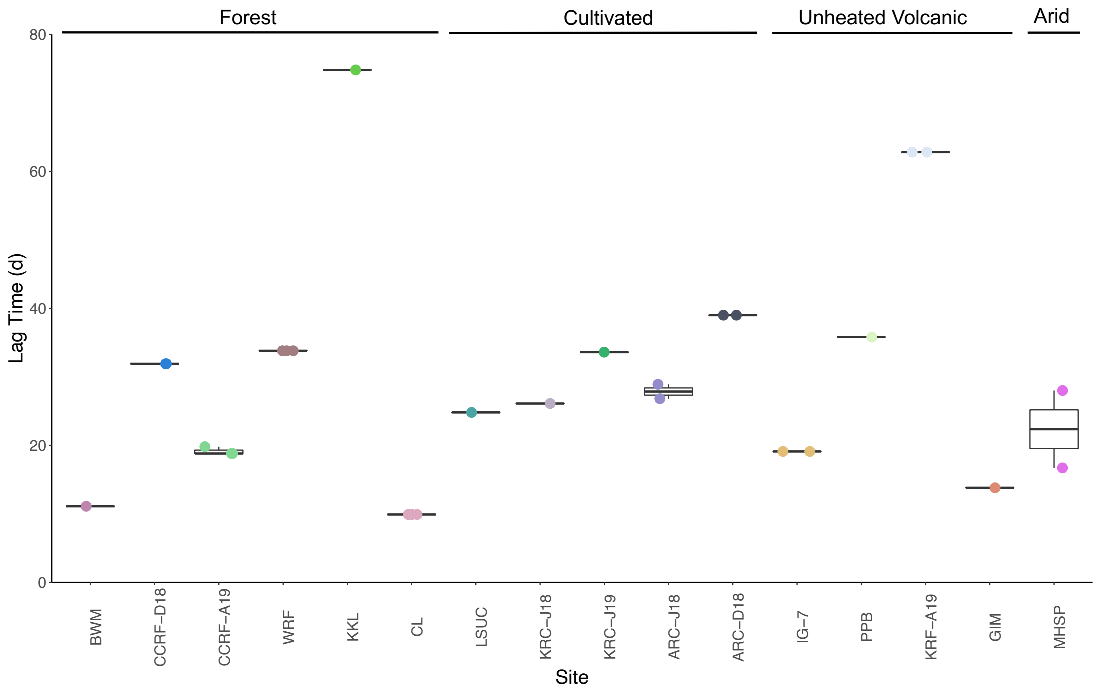

Figure S1B.

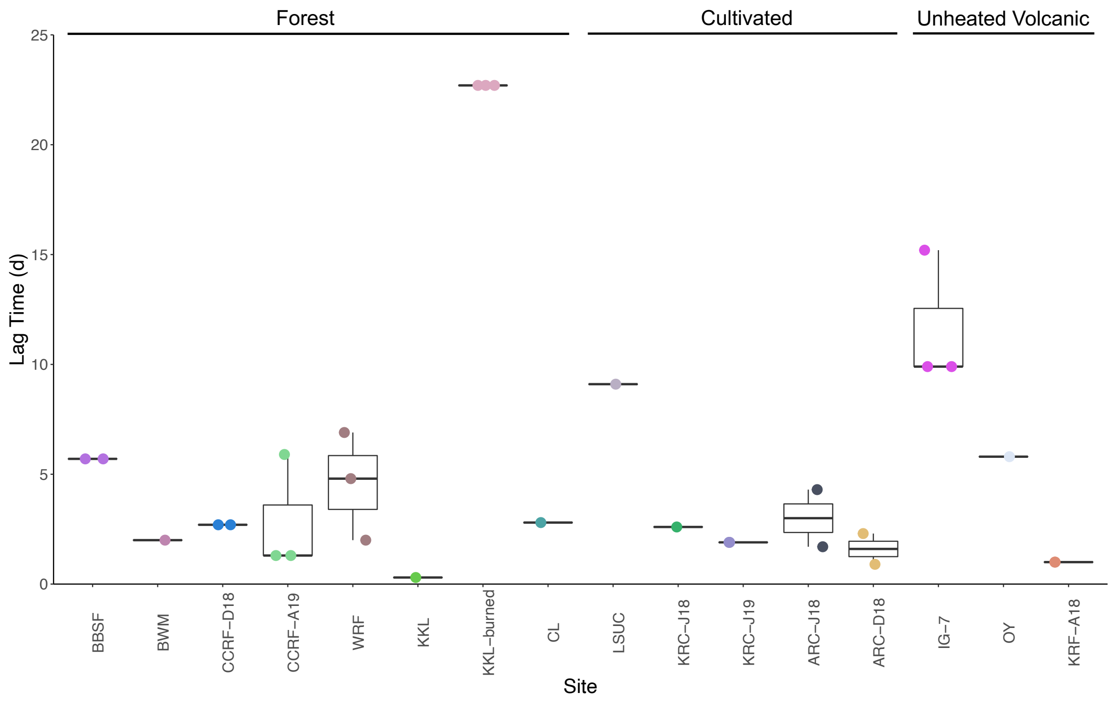

Figure S2. Apparent lag times (d) for flooded soils and unvegetated sediment sites. Lag times are shown at 25 ºC (A) and at 60 ºC (B). Median values are indicated by solid bars.

Figure S2A.

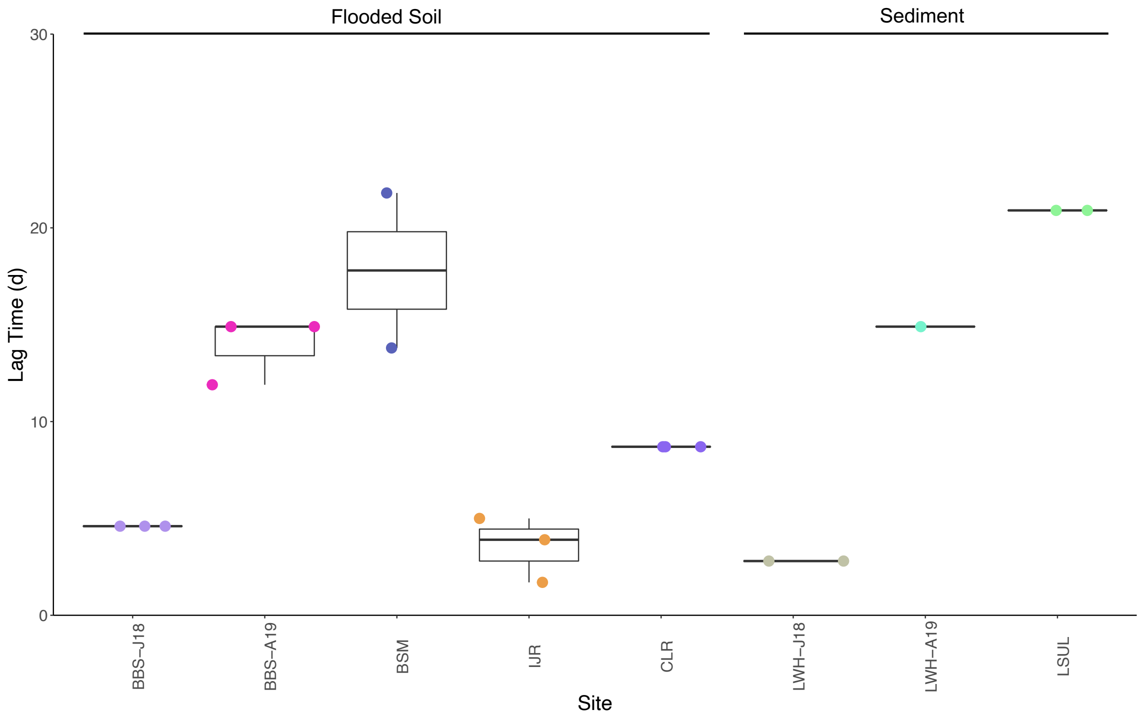

Figure S2B.

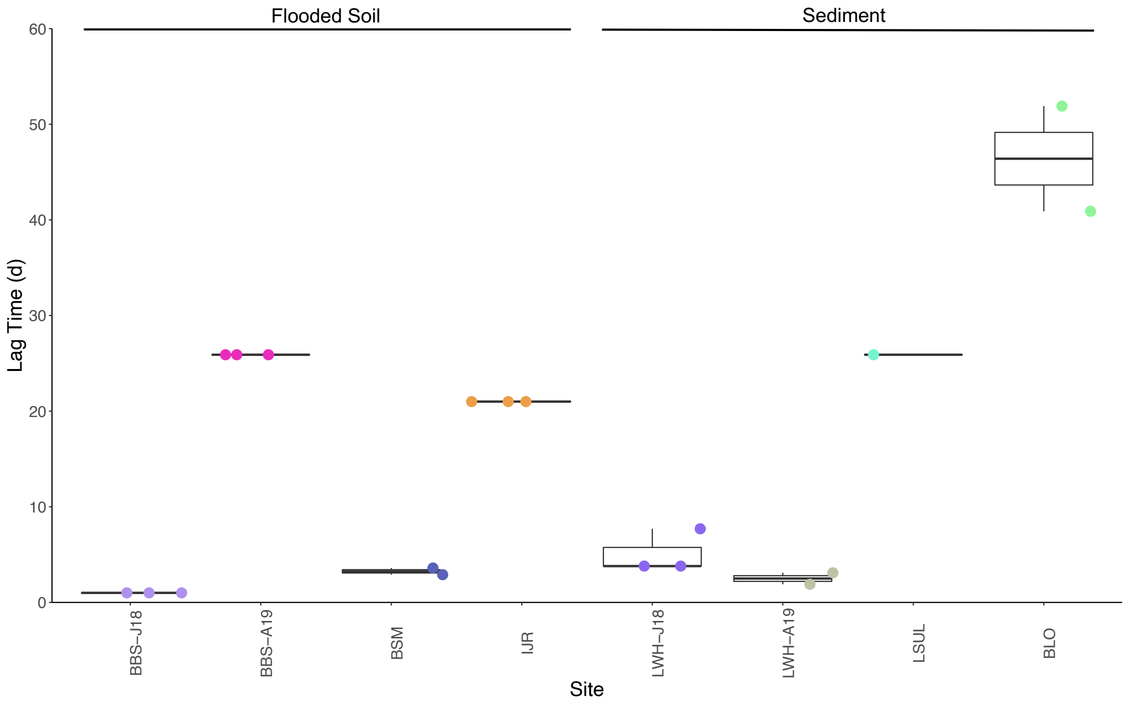

Table S1. Names, abbreviations, and GPS coordinates for soil, sediment, and hot springs sites.

Site	Acronym	GPS coordinates
**Forest Soil**		
Bluebonnet Swamp forest, mixed hardwood forest, Louisiana, USA	BBSF	30.37188 -91.10026
Baker Wood, mixed hardwood, coniferous forest, Maine, USA	BWM	44.10667, -69.17897
Mesquite stand, *Prospio glandulosa*, Death Valley, California, USA	MSDV	36.48528, -116.87823
Chain of Craters Road Forest, December 2018, Hawai`i, USA	CCRF-D18	19.38740, -155.02910
Chain of Craters Road Forest, April 2019, Hawai`i, USA	CCRF-A19	19.39160, -155.24533
Wright Road Forest, Volcano, Hawai'i, USA	WRF	19.45410, -155.24304
Kipukakulalio *Acacia koa*, Mauna Loa, Hawai'i, USA	KKL	19.47418, -155.37173
Kipukakulalio *Acacia koa*, burned, Mauna Loa, Hawai'i, USA	KKL-burned	19.47531, -155.35993
Puhimau Geothermal Area site A, December 2018, Hawai'i, USA	PGA-D18	19.38935, -155.24857
Puhimau Geothermal Area site A, December 2019, Hawai'i, USA	PGA-D19	19.38930, -155.24849
Kilauea Volcano, Pu'u Puai, closed canopy forest stand	PPC	19.41704, -155.25510
Miyaka-jima *Castenopsis seiboldii* Forest, Japan	CL	34.1119, 139.50150
**Cultivated Soil**		
LSU Campus cultivated soil, Louisiana, USA	LSUC	30.41329, -91.16923
Kueny Ranch cultivated soil, Fields, Oregon, July 2018	KRC-J18	42.43294, -118.62598
Kueny Ranch cultivated soil, Fields, Oregon, July 2019	KRC-J19	42.432945, -118.62599
Amauulu Road cultivated soil, July 2018, Hawai`i, USA	ARC-J18	19.72886, -155.10660
Amauulu Road cultivated soil, December 2018, Hawai`i, USA	ARC-D18	19.72807, -155.11127
**Geothermally-heated Volcanic Soil**		
Kilauea Volcano Sulphur Bank site A	KSB-A	19.43225, -155.26117
Kilauea Volcano Sulphur Bank site B	KSB-B	19.43213, -155.2612
Puhimau Geothermal Area site B	PGB	19.38921, -155.24849
Puhimau Geothermal Area site C-1	PGC-D18	19.38909, -155.24999
Puhimau Geothermal Area site C-2	PGC-A19	19.38903, -155.25008
Miyake-jima Igaya-7	IG-7	34.08940, 139.51410
Miyake-jima Oyama summit	OY	34.08020, 139.51900
Kilauea Volcano Pu'u Puai Bare	PPB	19.40653, -155.25726
Kilauea Volcano, Manua Ulu	KMU	19.33935, -155.20477
Kilauea Volcano, Hilina Pali	KHP	19.34033, -155.27530
Krafla, Iceland, August 2018	KRF-A18	65.71794, -16.78518
Krafla, Iceland, August 2019	KRF-A19	65.71736, -16.78603

Grímsey Island Meadow	GIM	66.56422, -18.01805
**Arid Soil**		
Mickey Hot Springs Unheated	MHSU	42.67857, -118.34825
Mickey Hot Springs Playa	MHSP	42.67538, -118.34673
Kueny Ranch Uncultivated	KRU	42.43263, -118.62647
Alvord Basin Pan, Borax-1	ABPB1	42.32649, -118.60987
Alvord Basin Pan, Borax-2	ABPB2	42.32758, -118.60500
Hastings Cutoff Utah	HCU	40.98092, -113.98805
**Flooded Soil**		
Bluebonnet Swamp, June 2018. Louisiana, USA	BBS-J18	30.36983, -91.10767
Bluebonnet Swamp, April 2019, Louisiana, USA	BBS-A19	30.37077, -91.10785
Baker Swamp, Maine, USA	BSM	44.10675, -69.179578
Ibaraki University rice plot, Ami-machi, Japan	IJR	36.03548, 140.21423
Experimental rice field, Crowley, Louisiana, USA	CLR	30.24693, -92.34631
**Sediment**		
Lake Waiau, June 2018, Hawai`i, USA	LWH-J18	19.81138, -155.47756
Lake Waiau, Apr 2019, Hawai`i, USA	LWH-A19	19.8115, -155.47707
LSU Lake, Baton Rouge, Louisiana, USA	LSUL	30.40922, -91.16472
Borax Lake, Oregon, outflow sediment	BLO	42.32755, -118.60149
Uxahryggjavegur, Iceland, 2nd order stream	UXA	64.45276, -21.10985
**Hot Springs**		
Mickey Hot Springs, July 2018, 35 ºC	MHS-J18-35	42.67722, -118.34640
Mickey Hot Springs, July 2018, 60 ºC	MHS-J18-60	42.67730, -118.34685
Mickey Hot Springs, July 2018, 86 ºC	MHS-J18-86	42.67755, -118.34700
Mickey Hot Springs, July 2019, 25 ºC	MHS-J19-25	42.67735, -118.34639
Mickey Hot Springs, July 2019, 60 ºC	MHS-J19-60	42.67733, -118.34683
Mickey Hot Springs, July 2019, 69 ºC	MHS-J19-69	42.67716, -118.34677
Alvord Hot Springs, 30 ºC	AHS-30	42.54419, -118.53393
Alvord Hot Springs, 60 ºC	AHS-60	42.54419, -118.53394
Alvord Hot Springs, 70 ºC	AHS-74	42.54418, -118.53394
Borax Hot Springs, 46 ºC	BHS-46	42.33417, -118.60302
Borax Hot Springs, 60 ºC	BHS-60	42.33417, -118.60302

Table S2. Physical characteristics of soils; all values are means ± 1 standard error. Temperature in ºC and water content as g gdw^{-1}.

Site	pH	OM (%)	Temperature	Water Content
**Forest Soil**				
Bluebonnet Swamp Forest (BBSF)	5.4 ± 0.95	6.0 ± 0.1	30	0.78 ± 0.04
Baker Wood, Maine (BWM)	5.5 ± 0.03	17.6 ± 1.6	19.4	0.63 ± 0.02
Mesquite Stand Death Valley (MFDV)	3.2 ± 0.10	3.2 ± 0.1	43.2	0.99 ± 0.001
Chain of Craters Road Forest, Dec 2018 (CCRF-D18)	5.2 ± 0.07	23.3 ± 2.8	17.3	0.67 ± 0.05
Chain of Craters Road Forest, Apr 2019 (CCRF-A19)	5.2 ± 0.07	24.2 ± 1.7	18.6	0.67 ± 0.05
Wright Road Forest (CCRF-A19)	4.5 ± 0.14	51.9 ± 3.0	19.5	0.24 ± 0.01
Kipukakulalio *Acacia koa* (KKL)	5.5 ± 0.04	31.0 ± 1.2	16.8	0.61 ± 0.03
Kipukakulalio *Acacia koa*-burned (KKL-burned)	5.82 ± 0.06	42.9 ± 9.87	24.7	0.75 ± 0.80
Puhimau Geothermal Area A, Dec 2018 (PGA-D18)	5.8 ± 0.04	1.7 ± 0.1	24.5	0.95 ± 0.01
Puhimau Geothermal Area A, Apr 2019 (PGA-A19)	5.8 ± 0.04	1.7 ± 0.1		0.90 ± 0.03
Kilauea Pu`u Pua`i Canopy (PPC)	4.1 ± 0.17	96.8 ± 0.9	18.1	0.18 ± 0.01
Miyake-jima forest (CL)	5.2 ± 0.05	21.8 ± 0.5	24.1	0.56 ± 0.01
**Cultivated Soil**				
LSU-cultivated (LSUC)	5.7 ± 0.06	4.1 ± 0.1	34.8	0.85 ± 0.0
Kueny Ranch Cultivated, Jun18 (KRC-J18)	7.1 ± 0.08	6.8 ± 0.3		0.82 ± 0.01
Kueny Ranch Cultivated, Jul19 (KRC-J19)	7.1 ± 0.08	5.5 ± 0.2	29.2	0.92 ± 0.01
Amauulu Road Cultivated, Jul 2018 (ARC-J19)	5.6 ± 0.11	29.7 ± 0.8	33.8	0.57 ± 0.01
Amauulu Road Cultivated, Dec 2018 (ARC-D18)	5.6 ± 0.11	31.6 ± 0.6	27.1	0.59 ± 0.01
**Geothermally-heated Volcanic Soil**				
Kilauea Sulphur Bank A (KSB-A)	2.6 ± 0.09	11.2 ± 1.3	93	0.70 ± 0.01
Kilauea Sulphur Bank B (KSB-B)	3.8 ± 0.11	9.5 ± 0.3	25.6	0.71 ± 0.02
Puhimau Geothermal Area B (PGB)	6.6 ± 0.07	6.9 ± 1.5	63	0.74 ± 0.10
Puhimau Geothermal Area C, Dec 2018 (PGC-D18)	6.7 ± 0.04	11.3 ± 1.3	83	0.58 ± 0.05
Puhimau Geothermal Area C, Apr 2019 (PGC-A19)	6.7 ± 0.04	11.3 ± 1.3	81	0.44 ± 0.11
**Unheated Volcanic Soil**				
Miyake-jima Igaya-7 (IG-7)	4.5 ± 0.03	1.7 ± 0.1	22.1	0.81 ± 0.02
Miyake-jima summit (OY)	4.8 ± 0.05	3.1 ± 0.4	22.4	0.84 ± 0.0
Kilauea Pu`u Pua`i Bare (PPB)	5.6 ± 0.04	1.4 ± 0.3	25.4	0.90 ± 0.03
Kilauea Mauna Ulu (KMU)	5.3 ± 0.03	0.8 ± 0.1	34.8	0.99 ± 0.0
Kilauea Hilina Pali (KHP)	5.1 ± 0.08	2.1 ± 0.4	39.4	0.98 ± 0.0

Krafla Geothermal Area, Iceland, Aug 2018 (KRF-A18)	6.2 ± 0.06	16.3 ± 1.5	10.1	0.59 ± 0.02
Krafla Geothermal Area, Iceland, Aug 2019 (KRF-A19)	6.2 ± 0.06	9.3 ± 0.4	9.7	0.66 ± 0.01
Grímsey	5.8 ± 0.07	8.7 ± 0.4	7.7	0.77 ± 0.02
**Arid Soil**				
Mickey Hot Springs unheated soil (MHSU)	9.7 ± 0.07	3.5 ± 0.3		0.93 ± 0.01
Mickey Hot Springs playa soil (MHSP)	9.9 ± 0.12	3.0 ± 0.1	43	0.99 ± 0.002
Kueny Ranch Uncultivated (KRU)	7.0 ± 0.12	6.1 ± 0.2	54.2	0.94 ± 0.01
Alvord Basin Pan, Borax-1 (ABPB1)	9.3 ± 0.02	18.3 ± 0.3	28.1	0.48 ± 0.03
Alvord Basin Pan, Borax-2 (ABPB2)	9.8 ± 0.08	11.4 ± 0.1	26.7	0.65 ± 0.01

Table S3. Physical characteristics of sediments and flooded soils; all values are means ± 1 standard error. Temperature in ºC and water content as g gdw^{-1}.

Site	pH	OM (%)	Temperature	Water Content
**Flooded Soil**				
Bluebonnet Swamp Jun 2018 (BBS-J18)	5.8 ± 0.08	27.8 ± 0.7	27.9	0.11 ± 0.005
Bluebonnet Swamp, Apr 2019 (BBS-A19)	5.8 ± 0.08	25.9 ± 1.1	18.2	0.19 ± 0.02
Baker Swamp, Maine (BSM)	5.2 ± 0.05	53.3 ± 2.1	18.2	0.35 ± 0.09
Ibaraki, Japan Rice (IJR)	5.2 ± 0.12	9.6 ± 0.3	13.1	0.61 ± 0.02
Crowley, Louisiana Rice (CLR)	6.4 ± 0.12	3.9 ± 0.1	21.3	0.76 ± 0.01
**Sediment**				
Lake Waiau, Hawai`i June 2018 (LWH-J18)	7.2 ± 0.07	4.5 ± 0.1	9.2	0.47 ± 0.003
Lake Waiau, Hawai`i Apr 2019 (LWH-A19)	7.2 ± 0.07	9.8 ± 4.4	6.5	0.51 ± 0.08
LSU Lake (LSUL)	8.3 ± 0.26	0.2 ± 0.05	28.5	0.84 ± 0.001
Borax Lake, Oregon (BLO)	7.3 ± 0.02	8.3 ± 0.4	23.5	0.52 ± 0.07
Uxahryggjavegur, Iceland (UXA)	--	13.9 ± 0.3	9.1	0.44 ± 0.02

Table S4. Physical characteristics of hot spring sediments; all values are means ± 1 standard error. Temperature in ºC and water content as g gdw^{-1}.

Site	pH	OM (%)	Temperature	Water Content
MHS-J18 35	--	8.0 ± 0.3	35	0.46 ± 0.03
MHS-J18 60	--	4.3 ± 0.3	60	0.57 ± 0.02
MHS-J18 86	--	4.7 ± 0.4	86	0.55 ± 0.04
MHS-J19 25	8.3 ± 0.12	10.2 ± 1.0	25.2	0.34 ± 0.06
MHS-J19 60	9.0 ± 0.08	4.2 ± 0.4	60	0.36 ± 0.08
MHS-J19 69	7.3 ± 0.03	4.9 ± 0.2	69.2	0.44 ± 0.08
AHS 30	8.0 ± 0.05	7.9 ± 0.4	30	0.27 ± 0.02
AHS 60	7.2 ± 0.32	9.5 ± 0.7	60	0.20 ± 0.05
AHS 70	7.5 ± 0.28	4.5 ± 0.3	74	0.53 ± 0.01
BHS-46	7.3 ± 0.03	4.1 ± 1.5	45.7	0.34 ± 0.04
BHS-60	7.3 ± 0.02	8.3 ± 0.4	60.5	0.55 ± 0.13
